# A Study about Regioisomeric Hydroquinones with Multiple Intramolecular Hydrogen Bonding

**DOI:** 10.3390/molecules22040593

**Published:** 2017-04-07

**Authors:** Maximiliano Martínez-Cifuentes, Wilson Cardona, Claudio Saitz, Boris Weiss-López, Ramiro Araya-Maturana

**Affiliations:** 1 Programa Institucional de Fomento a la Investigación, Desarrollo e Innovación, Universidad Tecnológica Metropolitana, Ignacio Valdivieso 2409, Casilla 9845, Santiago 8940577, Chile; 2 Departamento de Ciencias Químicas, Facultad de Ciencias Exactas, Universidad Andrés Bello, Autopista Concepción-Talcahuano 7100, Talcahuano 4300866, Chile; wcardona@unab.cl; 3 Departamento de Química Orgánica y Fisicoquímica, Facultad de Ciencias Químicas y Farmacéuticas, Universidad de Chile, Santos Dumont 964, Casilla 233, Santiago 8380494, Chile; clsaitz@ciq.uchile.cl; 4 Departamento de Química, Facultad de Ciencias, Universidad de Chile, Las Palmeras 3425, Casilla 653, Santiago 7800003, Chile; bweiss@uchile.cl; 5 Instituto de Química de Recursos Naturales, Universidad de Talca, Av. Lircay s/n, Casilla 747, Talca 3460000, Chile

**Keywords:** hydroquinone, hydrogen bond, DFT, NBO, AIM

## Abstract

A theoretical exploration about hydrogen bonding in a series of synthetic regioisomeric antitumor tricyclic hydroquinones is presented. The stabilization energy for the intramolecular hydrogen bond (IHB) formation in four structurally different situations were evaluated: (a) IHB between the proton of a phenolic hydroxyl group and an *ortho*-carbonyl group (forming a six-membered ring); (b) between the oxygen atom of a phenolic hydroxyl group and the proton of an hydroxyalkyl group (seven membered ring); (c) between the proton of a phenolic hydroxyl group with the oxygen atom of the hydroxyl group of a hydroxyalkyl moiety (seven-membered ring); and (d) between the proton of a phenolic hydroxyl group and an oxygen atom directly bonded to the aromatic ring in *ortho* position (five-membered ring). A conformational analysis for the rotation around the hydroxyalkyl substituent is also performed. It is observed that there is a correspondence between the conformational energies and the IHB. The strongest intramolecular hydrogen bonds are those involving a phenolic proton and a carbonyl oxygen atom, forming a six-membered ring, and the weakest are those involving a phenolic proton with the oxygen atom of the chromenone, forming five-membered rings. Additionally, the synthesis and structural assignment of two pairs of regioisomeric hydroquinones, by 2D-NMR experiments, are reported. These results can be useful in the design of biologically-active molecules.

## 1. Introduction

Hydrogen bonding (HB) plays a central role determining the structure, properties, and functions of biologically-important molecules [1,2,3]. HB interaction is the driving factor in many chemical and biochemical processes. Thus, studying HB in model compounds, which are structurally related to molecules fundamental for life, can be useful for the design of new biologically-active molecules. An intramolecular hydrogen bond (IHB) is formed when the proton donor and acceptor are in the same molecule [4]. An intermolecular hydrogen bond (IEHB) is formed when the donor and acceptor are in different molecules and, unlike IHB, IEHB energy is obtained easily from the energy difference between the two molecules together and separated. The non-bonded situation for IHB is not as easy to assess [5]. The identification of the close contact of an attractive IHB can sometimes be ambiguous, for example, those bonds found in a crystal. Experimental and theoretical tools, such as NMR and quantum chemical calculations, have been employed to evaluate the existence and strength of this kind of interaction in a series of tricyclic antitumor hydroquinones [5,6,7,8,9].

*p*-Hydroquinones (*p*-HQ) and their oxidized form, *p*-quinones (*p*-Q) are naturally occurring compounds, commonly isolated from different living organisms [10,11,12,13,14,15]. The biological activity of *p*-HQs has been related to their capability to form the semiquinone radical, through a one-electron oxidation. These intermediates have been associated to biological properties, such as pro-oxidant activity, by interacting with several intracellular molecules, such as DNA and proteins [16,17,18]. For example, in the inhibitors of mitochondrial complex I (NADH:ubiquinone oxidoreductase), The active compounds usually exhibit aromatic rings with a hydroquinone/quinone motif, planarity being one of the geometrical requirements. Three classes of inhibitors share a common binding domain in mitochondrial complex I (NADH:ubiquinone oxidoreductase), [19] and its interaction with complex I is highly sensitive to structural modifications [20]. In effect, small structural changes on hydroquinone scaffolds can determine the complex I inhibition or uncoupling of tumoral oxidative phosphorylation [21].

Modulation of the electron-transfer capability in *p*-HQs and *p*-Qs is an important variable to explore in order to obtain new biologically active compounds. Among the interactions that play a central role in this issue, the formation of inter- or intramolecular hydrogen bonds is of central importance in these molecules [22,23]. A recent study about the electrochemistry of hydroxy quinones possessing intramolecular hydrogen bonds (IHBs) shows that this interaction stabilizes the anion radical structure, leading to a shift in reduction potentials toward less negative values, making the process more spontaneous, when compared with hydroxy-quinones without IHBs [24]. IHBs have shown appreciable effects on the electrochemistry [25] and the antioxidant properties of hydroquinone and related phenols [26]. Antioxidant properties and free radical scavenging reactivity of a family of hydroxynaphthalenones and dihydroxyanthracenones were previously reported [27].

**Figure 1:**
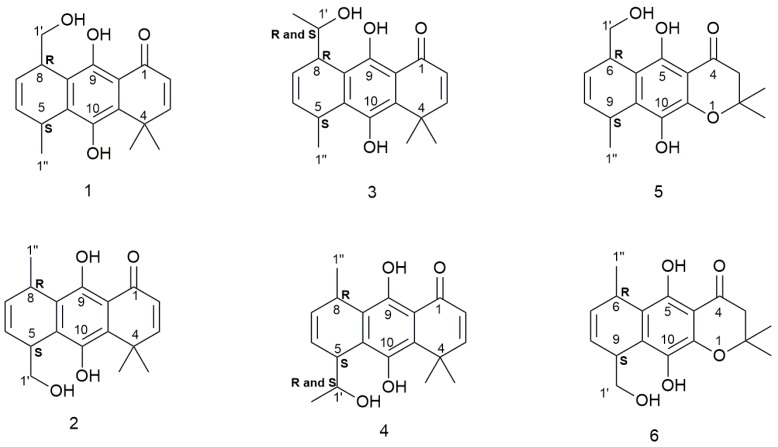
Structure of molecules studied in this work.

The *o*-carbonyl hydroquinone moiety is an important structural feature of several natural products with different biological activities, such as doxorubicin, daunorubicin, [28] acylnaphthohydroquinone [29,30], shikonin [30], and peyssonol A [31]. In previous works, we have shown that antioxidant molecules, containing an *o*-carbonyl hydroquinone moiety, inhibit mitochondrial tumor cell respiration [32,33,34]. Theoretical and biochemical insights about the activity of these hydroquinones have been recently described highlighting the role of intramolecular hydrogen bonding [35]. In this work, we carry out a conformational study of previously-reported hydroquinones **1**,**2** [36] and some new hydroquinone analogues, **3**–**6**, (Figure 1) analyzing the different types of intramolecular hydrogen bonding (IHB) displayed and their influences on the conformational preferences of these molecules. An experimental (NMR) and a theoretical approach (MO) are employed in this report. The IHBs are studied by natural-bond-orbital (NBO) and atoms-in-molecules (AIM) analyses. Hydroquinone **3** was theoretically examined using diastereomers (5*S*, 8*R*, 1′*S*, and 1′*R*) and **4** was studied using diastereomers (5*S*, 8*R*, 1′*S*, and 1′*R*). The synthetic route used for obtaining compounds **1** and **2** was tested to know if compounds **3**–**6** are accessible experimentally. In effect, compounds **3**–**6** are obtained using the same methodology, though the respective isolated compounds were not stereochemically assigned.

## 2. Results

### 2.1. Synthesis

**Scheme 1:**
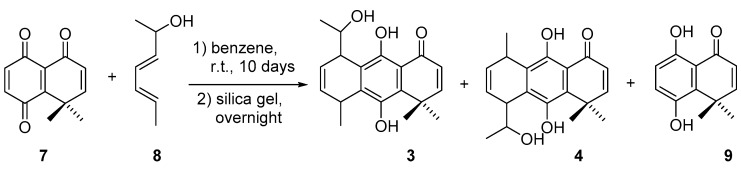
Synthesis of compounds **3** and **4**.

In order to test if the synthetic route used in the synthesis of hydroquinones **1** and **2** is also adequate when a secondary dienol is used, a Diels-Alder reaction between quinone **7** and racemic (3*E*,5*E*)-hepta-3,5-dien-2-ol (**8**) (Figure 1, Scheme 1) was carried out. Though diasteromeric mixtures of regioisomers **3** and **4** must be obtained, only two major compounds were isolated. However, the stereochemical assignments of the isolated diasteromers were not carried out. Compounds **3** and **4** were obtained at 26% yield and 18% yield, respectively, in addition to hydroquinone **9** at 47% yield. The percentage of **9**, a hydroquinone reduction product of **7**, is very high when compared with the yield obtained with the primary dienol (15% yield) [37]. Racemic diene **8** was obtained by the reaction of hexadienal and methylmagnesium bromide.

**Scheme 2:**
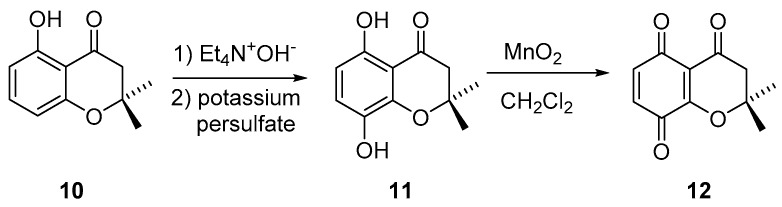
Synthesis of dienophile **12**.

Dienophile **12** was synthesized following a described route starting from compound **10** (Scheme 2) [38].

**Scheme 3:**
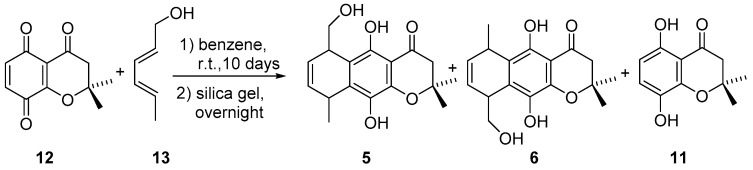
Synthesis of compounds **5** and **6**.

Hydroquinones **5** and **6** were obtained by the same synthetic route used for compounds **3** and **4**, a Diels-Alder reaction was performed between 2,2-dimethyl(2*H*)-chromen-4,5,8(3*H*)-trione (**12**) and (2*E*,4*E*)-hexa-2,4-dien-1-ol (**13**), followed by enolization with silica gel (Scheme 3). This method allowed obtaining compounds **5** and **6** in 39% yield and 20% yield, respectively. Additionally, hydroquinone **11** was obtained with 36% yield.

### 2.2. Structural Assignments

**Figure 2:**
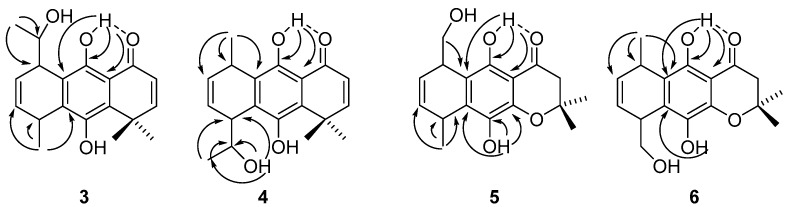
Key HMBC long-range correlations used for structural assignments of regioisomeric compounds.

Tricyclic hydroquinones **3**–**6** are closely related to the structure of a series of previously-studied tricyclic regioisomeric antitumor hydroquinones [32,34]. An *ortho*-carbonyl substituted hydroquinone derivative is an anticancer agent that acts by inhibiting mitochondrial bioenergetics and by inducing G2/M-phase arrest in mouse mammary adenocarcinoma TA3/Ha [21]. The complete assignments of the ^13^C resonances by concerted use of Heteronuclear Multiple-Quantum Correlation (HMQC), Heteronuclear Multiple Bond Correlation (HMBC), and theoretical calculations have been previously reported [36,39]. Intramolecular hydrogen bonds in some of these *o*-carbonyl hydroquinones have been recently studied [40]. The key feature, in which NMR assignments are based, is the different nature of the phenolic hydroxyl groups. The hydroxyl group at C-9 is engaged in a strong IHB with the carbonyl oxygen atom, and this is evidenced by its downfield resonance at δ ranging from 11.34 to 13.17. The non-chelated phenolic hydroxyl protons, from compounds **1**–**4**, resonate between 4.35 and 8.02 ppm. The non-chelated phenolic protons exhibit a chemical shift concentration-dependent behavior, because of the intermolecular interactions. The 10-OH resonances for compounds **5** and **6** are 5.05 and 5.70 ppm, respectively. Figure 2 shows the main HMBC correlations used for the assignments of hydroquinones **3**–**6**.

### 2.3. Conformational Analysis

**Figure 3:**
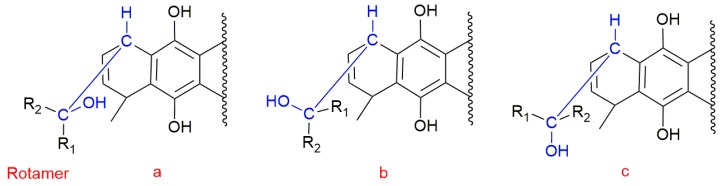
Rotamers generated by rotation of dihedral angle H8(5)-C8(5)-C1′R1R2-OH (molecules **1** to **4**) or H9(6)-C9(6)-C1′R1R2-OH (molecules **5** and **6**).

The conformational flexibility of these structures arises from the rotation of CH-CHR-OH fragment (R = H for **1**, **2**, **5**, **6**, and R = CH_3_ for **3** and **4**). In order to analyze the conformational preferences of the studied molecules, we searched for minima rotating the dihedral angle H8(5)-C8(5)-C1′HR-OH (**1** to **4**) or H9(6)-C9(6)-C1′HH-OH (**5** and **6**), as shown in Figure 3.

**Figure 4:**
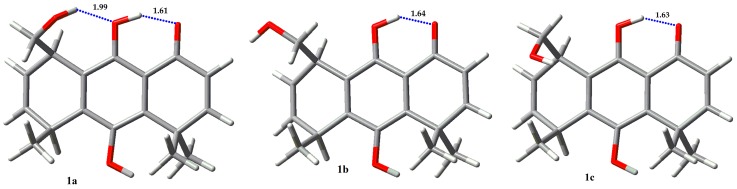
Rotamers for **1**.

**Figure 5:**
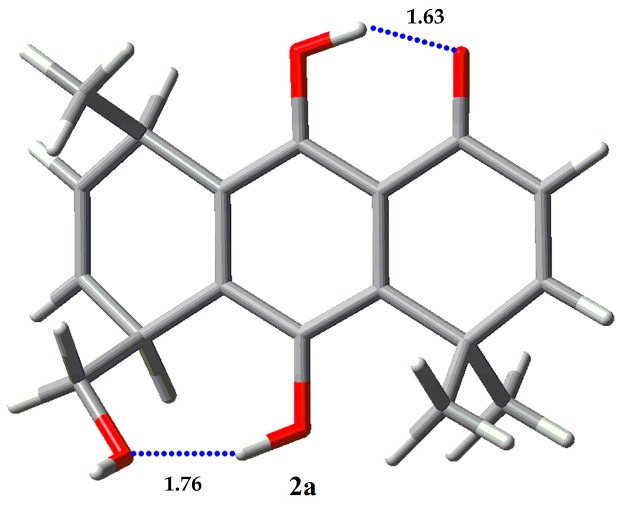
Rotamers for **2**.

The hydroxymethyl group in compounds **1** (Figure 4, Table 1) and **2** (Figure 5, Table 2) exhibits a markedly different conformational behavior. When it is bonded to C-5 (compound **2**) it exists as a single rotamer around the C5-CH_2_OH single bond, as evidenced by the very different values of the vicinal coupling constants of the methylene protons with HC-5, about 10 and 3 Hz, assigned to the anti and gauche protons, respectively. Their preferred conformation may be a consequence, in turn, of the preferred conformation of the hydroxyl group bonded to C-10, whose proton is oriented towards the *gem*-dimethyl group, thus avoiding the repulsion between the non-bonded electrons of oxygen atoms and the alkyl groups. Theoretical calculations support these considerations showing that this is the only conformation populated at room temperature. Compound **2** mainly shows two intramolecular hydrogen bonds, in which one of the phenolic protons are bonded to the carbonyl forming a six-membered ring, and the other is hydrogen bonded to the hydroxymethyl oxygen atom forming a seven-membered ring [36].

In compound **1**, with the hydroxymethyl group at C-8, instead of C-5, the vicinal coupling constant exhibits values in the range 4–6 Hz, evidencing the presence of a mixture of rotamers. This fact shows that the hydrogen bond between a phenolic proton and the oxygen atom from the carbinol group, as observed for compounds **2**, **4**(*R*), and **4**(*S*), is stronger than those bonded through a carbinol proton and a phenolic oxygen atom (compounds **1**, **3**(*R*), and **3**(*S*)).

**Figure 6:**
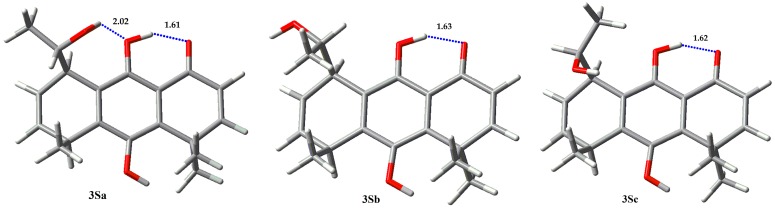
Rotamers for **3*S***.

**Figure 7:**
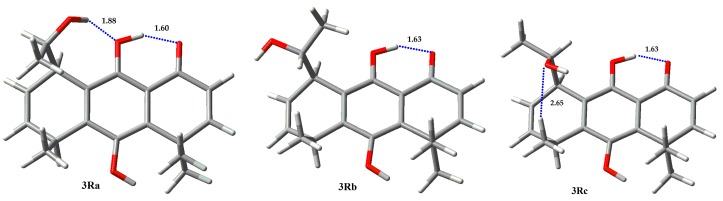
Rotamers for **3*R***.

**Figure 8:**
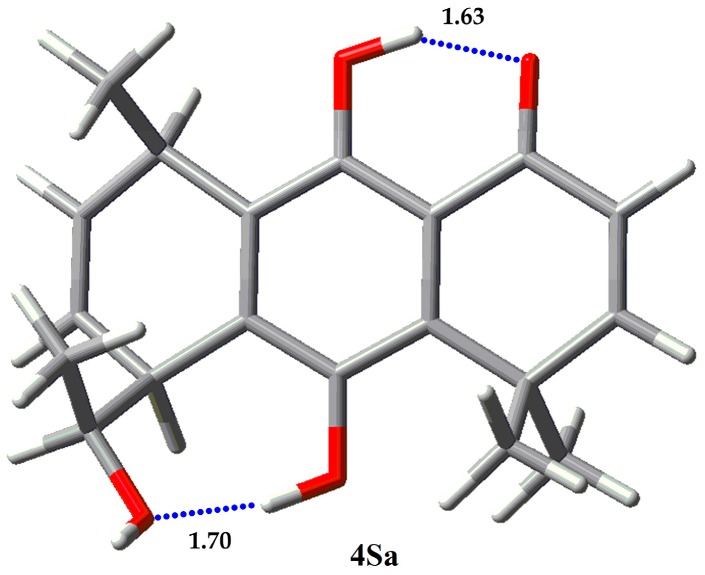
Single rotamer for **4*S***.

**Figure 9:**
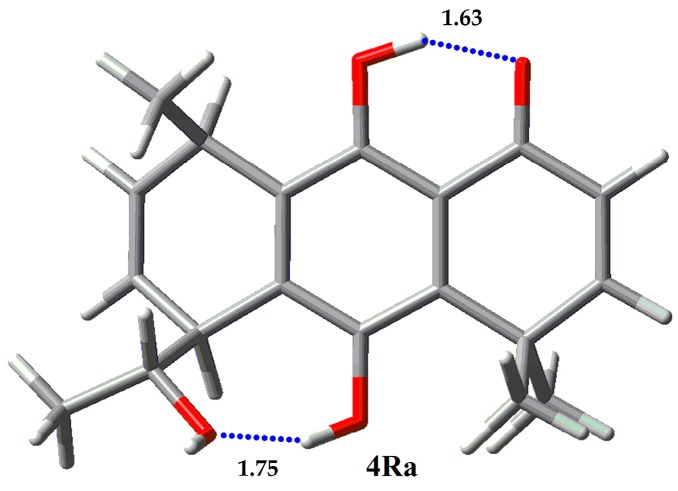
Single rotamer for **4*R***.

However, calculations on compound **3** show a markedly different behavior between epimers *S* (Figure 6, Table 3) and *R* (Figure 7, Table 4) at carbinolic carbon. The analysis of the **3*S*** rotamers show that **3*S*a** exhibits a hydrogen bond between the phenolic oxygen atom and the proton of the secondary hydroxyl group, and it has a population higher than 95% at 298 K. On the other hand, **3**(*R*) conformers show very different results, where the **3*R*c** rotamer is the most stable one, with a 77% population, but without hydrogen bonding between the hydroxyalkyl substituent and the phenolic hydroxyl group, because that interaction imposes a steric repulsion between the methyl group, located at C5, and the methyl group of the hydroxyethyl substituent (1,4-diaxial repulsion in the boat like conformation of cyclohexane). The lower energy of the **3*R*c** conformer relies on the non-conventional stabilizing interaction between the hydroxyl oxygen atom and the methyl protons. Secondary alcohol analogs of **4** (epimers *R* and *S* at carbinolic center) show a similar conformational behavior, despite their absolute configuration (Figure 8 and Figure 9, Table 5 and Table 6).

**Figure 10:**
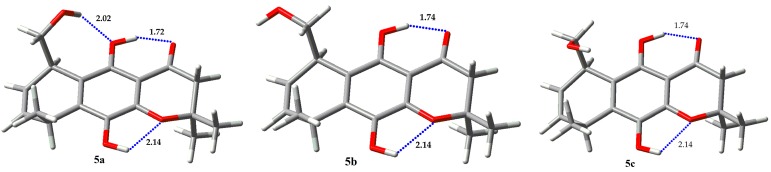
Rotamers for **5**.

**Figure 11:**
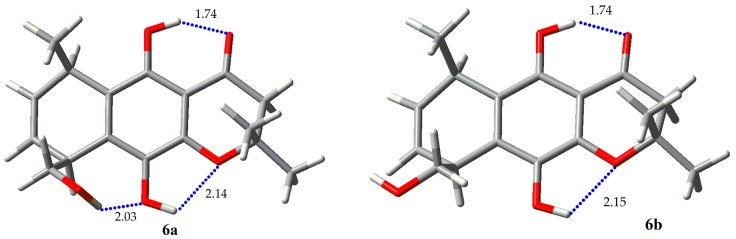
Rotamers for **6**.

A different behavior takes place in chromenone derivatives **5** (Figure 10, Table 7) and **6** (Figure 11, Table 8). Compound **5** exhibits two hydrogen bonds in all calculated structures, the same already discussed before between one phenolic proton with the carbonyl oxygen atom forming a six membered ring, and a different hydrogen bond between the other phenolic proton and the oxygen atom of the chromenone ring, forming a five membered ring. The ^1^H-NMR spectrum evidences these interactions by displaying chemical shifts of phenolic protons at δ = 5.05 and 11.34 for OH-5 and OH-10, respectively. The conformation of the hydroxymethyl group here has a similar behavior to that described for compound **1**, displaying a mixture of three conformers. The preferred rotamer exhibits a hydrogen bond between the alkyl hydroxyl proton and the oxygen atom of the phenolic hydroxyl group. Compound **6** also exhibits the two hydrogen bonds of the phenolic protons described for regioisomer **5**. In this case, practically two rotamers of the hydroxymethyl group are present, though with the hydrogen-bonded structure of the hydroxymethyl group as the most populated one.

### 2.4. NBO and AIM Analysis

The NBO and AIM analysis of results for **1** and **2** are given in Table 9 and Table 10, respectively. The main rotamers for each molecule were studied (>5% population). The results of NBO analysis for molecule **1** shows that rotamer **1a** presents the highest values for the IHB O1^…^HO9 stabilization energy, compared with **1b** and **1c**. In addition, **1a** presents stabilization energy of 5.38 kcal/mol for the cooperative IHB O9^…^HO1′, not present in the other rotamers. Regioisomer **2** displays only one major populated rotamer, **2a**, which possess two IHB, O1^…^HO9 with 30.59 kcal/mol and O1′^…^HO10 with 16.26 kcal/mol. The strength of the last IHB is remarkably higher than the weakest IHB O9^…^H-O1′ present in **1a**, and it explains the difference in rotamer populations for compound **2** when compared with regioisomer **1**. The higher acidity of a phenolic proton, and the concomitant lower basicity of a phenolic oxygen compared with the corresponding properties of the proton and oxygen in a hydroxyl group bonded to an alkyl chain can explain these results.

AIM analysis shows negative values for both, the Laplacian of electron density at the critical point (∇^2^ρ) and the total electron energy density H (a.u.), which indicates the covalent nature of all IHB studied for **1** and **2**. Hydrogen bond energy (E_HB_) obtained from the potential energy density (V) as E_HB_ = (½)V [41] for rotamers of **1** and **2** are between 5.27 and 19.67 kcal/mol. The AIM E_HB_ values for the IHB O1^…^H-O9 present in all rotamers studied for **1** and **2** are around 19 kcal/mol, unlike NBO stabilization energies, which gives a value around 30 kcal/mol for the same interaction. The AIM E_HB_ values for the IHB O9^…^H-O1′ in **1a** (5.62 kcal/mol) is very similar to those obtained by NBO analysis (5.38 kcal/mol). On the other hand, the IHB O1′^…^H-O10 present in **2a** shows a significant difference between the AIM E_HB_ values (5.27 kcal/mol) and the NBO stabilization energy (16.26 kcal/mol).

NBO and AIM results for **3** and **4** are given in Table 11 and Table 12, respectively. Epimer **3*S*** presents a major rotamer **3*S*a** with a population higher than 95%. **3*S*a** has a high stabilization energy for the IHB O1^…^H-O9 (33.24 kcal/mol). Additionally, it has a stabilization energy of 4.61 kcal for the cooperative IHB O9^…^H-O1. On the other hand, epimer **3*R***, of this regioisomer, presents two main rotamers, **3*R*a** and **3*R*c**, populated with 17.85% and 77.28%, respectively. Though rotamer **3*R*a** presents two cooperative IHB with stabilization energies of 34.92 kcal/mol (O1^…^H-O9) and 8.50 kcal/mol (O9^…^H-O1′), it is less populated than **3*R*c**, which only presents one IHB of 30.73 kcal/mol. We were unable to find some stabilization energy between O1′ and H-C1′’ (distance of 2.65 Å) in the NBO calculations. This suggests that the lower population of rotamer **3*R*a** is due to the repulsive interaction between C5-methyl group and the methyl from the hydroxymethyl group at C8, in the boat-like conformation of the ring. Regioisomers **4*S*** and **4*R*** are present only in one main rotamer. **4*S*a** has two IHB, O1^…^H-O9 with 31.04 kcal/mol and O1′^…^H-O10 with 20.61 kcal/mol stabilization energies. The stabilization energies for **4*R*a** are 30.45 kcal/mol for O1^…^H-O9 and 15.67 kcal/mol for O1′^…^H-O10. The presence of these two strong IHB can explain the predominance of that rotamer for the two epimers.

Similar to what happened with **1** and **2**, molecules **3** and **4** also present negative values for both ∇^2^ρ and H, which indicates the covalent nature of all IHB studied for **3** and **4**. The strong IHB O1^…^H-O9 present differences between AIM E_HB_ values and the NBO stabilization energies (~19 vs. ~30 kcal/mol), similar to those observed in **1** and **2**. The IHB O9^…^H-O1′ present in **3Sa** and **3Ra** shows similar energy values from AIM and NBO analysis. The IHB O1′^…^H-O10 presents slightly lower values for AIM E_HB_ compared with the NBO stabilization energy.

For regioisomers **5** and **6** (Table 13), the analyses were carried out considering IHB O1^…^HO10 and not IHB O10^…^HO1′. The main rotamer of molecule **5a** presents three IHB: the strongest, O4…H-O5, with a stabilization energy of 21.57 kcal/mol, and the weakest, O1…H-O10, forming a five membered ring, stabilized by 1.07 kcal/mol, in addition to IHB O5^…^H-O1′ with a stabilization energy of 4.41 kcal/mol. The last is not present in the other rotamers and can account for the higher population of **5a** (48.44%). A comparison of the stabilization energies for **5b** and **5c** shows that both have approximately equal populations.

The most stabilized rotamer of molecule **6** (**6a**) also presents three IHB. The stabilization energies for the IHB are 19.86 kcal/mol for O4^…^H-O5, 4.40 kcal/mol for O6^…^H-O1′, and 1.16 kcal/mol for O1^…^H-O6. The other significant rotamer displays similar values for both IHB (19.88 and 1.06 kcal/mol), which indicates that the additional IHB present in **6a** accounts for its higher population.

AIM analysis reveals that rotamers of **5** and **6** present IHB of a covalent nature, similarly to the other studied molecules (Table 14). However, no bond critical points were found for IHB O1^…^H-O10, which was identified in the NBO analysis with stabilization energy around 1 kcal/mol (weak IHB). The IHB O4^…^H-O5, present in all rotamers studied for **5** and **6,** present a lower AIM E_HB_ value compared with their NBO stabilization energies.

From these results, it is clear that the strongest intramolecular hydrogen bonds are those involving a phenolic proton and carbonyl oxygen atom forming a six-membered ring, and the weakest are those involving a phenolic proton with the oxygen atom of the chromenone ring, which forms five-membered rings. An intermediate situation is observed with hydrogen bonds formed by protons of alkyl hydroxyl groups and phenolic oxygen atoms, forming seven-membered rings. The size of the ring in the last two hydrogen bonds appears to be a main factor determining the energetics of these IHB.

## 3. Materials and Methods

### 3.1. Synthetic Procedures

Melting points are uncorrected. All NMR spectra were acquired using a Bruker AVANCE DRX 300 spectrometer (Bruker BioSpin GmbH, Rheinstetten, Germany) operating at 300.13 MHz (^1^H) or 75.47 MHz (^13^C). Measurements were carried out at a probe temperature of 300 K.

Cycloadition of quinone **7** with (3*E*,5*E*)-hepta-3,5-dien-2-ol (**8**). A solution of quinone **7** (132 mg, 0.65 mmol) and diene **8** (105 mg, 0.94 mmol) in C_6_H_6_ (20 mL) was left at room temperature for ten days. A further addition of 2 g of silica gel and magnetic stirring overnight of the mixture allowed a solid mixture after filtration to be obtained, which was repeatedly washed with MeOH and the solvent removed. Column chromatography on silica gel with an 1:3 light petrol:EtOAc mixture allowed both hydroquinones to be separated. 9,10-Dihydroxy-8-(1-hydroxyethyl)-4,4,5-trimethyl-5,8-dihydroanthracen-1 (4*H*)-one (**3**), 67 mg (0.21 mmol, 32% yield): ^1^H-NMR δ (CDCl_3_): 1.31 (d, 3H, *J* = 6.9 Hz, 5-Me), 1.48 (d, 3H, *J* = 5.9 Hz, 8-CHOH-CH_3_), 1.54 (s, 3H, 4-Me), 1.58 (s, 3H, 4-Me), 2.35 (d, 1H, *J* = 3.1 Hz, OH), 3.68 (m, 1H, 8-H), 3.77 (m, 2H, 8-CHOH-CH_3_ and 5-H), 5.81 (dd, 1H, *J*_1_ = 5.5 Hz, *J*_2_ = 9.7 Hz, H-6), 6.16 (dd, 1H, *J*_1_ = 5.5 Hz, *J*_2_ = 9.7 Hz, 7-H), 6.23 (d, 1H, *J* = 10.1 Hz, H-2), 6.84 (d, 1H, *J* = 10.1 Hz, 3-H), 7.80 (s, 1H, 10-OH), 13.17 (s, 1H, 9-OH). ^13^C-NMR δ (CDCl_3_): 22.48 (5-Me), 23.04 (8-CHOH-CH_3_), 25.10 (4-Me), 25.49 (4-Me), 29.71 (C5), 38.38 (C4), 43.22 (C8), 76.60 (8-CHOH-CH_3_) 113.36 (C9a), 123.94 (C2), 124.58 (C6), 128.04 (C8a), 133.96 (C4a), 134.54 (C7), 135.59 (C10a), 144.82 (C10), 154.18 (C9), 192.00 (C1). IR (KBr) 3415.8, 2967.0, 1651.1 and 1451.9 cm^−1^. M.p. 221–223 °C. Anal. Found: C, 71.70; H, 7.02; Calc. for C_19_H_22_O_4_: C, 72.59; H, 7.05. 9,10-Dihydroxy-5-(1-hydroxyethyl)-4,4,8-trimethyl-5,8-dihydroanthracen-1(4*H*)-one (**4**), 45 mg (0.14 mmol, 22% yield): ^1^H-NMR δ (DMSO-*d*_6_): 1.18 (d, 3H, *J* = 6.4 Hz, 5-CHOH-CH_3_), 1.31 (d, 3H, *J* = 6.8 Hz, 8-Me), 1.57 (s, 3H, 4-Me), 1.58 (s, 3H, 4-Me), 3.48 (m, 1H, 5-H), 3.65 (m, 1H, 8-H), 4.13 (m, 1H, 5-CHOH-CH_3_), 4.28 (d, 1H, *J* = 6.1 Hz, OH), 5.90 (dd, 1H, *J*_1_ = 4.8 Hz, *J*_2_ = 10 Hz, 7-H), 6.00 (dd, 1H, *J*_1_ = 4.8 Hz, *J*_2_ = 10 Hz, 6-H), 6.22 (d, 1H, *J* = 10 Hz, H-2), 7.07 (d, 1H, *J* = 10 Hz, H-3), 7.80 (s, 1H, 10-OH), 13.36 (s, 1H, 9-OH). ^13^C-NMR δ (DMSO-*d*_6_): 21.98 (8-Me), 22.39 (5-CHOH-CH_3_), 25.09 (4-Me), 25.34 (4-Me), 30.65 (C8), 38.40 (C4), 41.81 (C5), 67.26 (5-CHOH-CH_3_), 112.32 (C9a), 123.53 (C2), 124.06 (C7), 124.78 (C8a), 132.67 (C6), 134.54 (C4a), 141.32 (C10a), 143.91 (C10), 154.02 (C9), 162.55 (C3), 191.46 (C1). IR (cm^−1^) 3291.5, 2956.5, 1619.8, 1471.1. M.p. 212–213 °C. Anal. Found: C, 72.53; H, 7.19. Calc. for C_19_H_22_O_4_: C, 72.59; H, 7.05.

Cycloadition of quinone **9** with (2*E*,4*E*)-hexa-2,4-dien-1-ol (**10**). A solution of quinone **9** (82 mg, 0.39 mmol) and diene **10** (38 mg, 0.38 mmol) in C_6_H_6_ (15 mL) was left at room temperature for ten days. A further addition of 2 g of silica gel and magnetic stirring overnight of the mixture allowed a yellow solid to be obtained after filtration, which was washed repeatedly with MeOH and the solvent removed. Column chromatography on silica gel with an 1:2 light petrol:EtOAc mixture allowed both hydroquinones to be separated. 5,10-dihydroxy-6-(hydroxymethyl)-2,2,9-trimethyl-2,3,6,9-tetrahydro-4*H*-benzo[*g*]chromen-4-one (**5**), 46.4 mg (0.15 mmol, 39% yield): ^1^H-NMR δ (CDCl_3_): 1.35 (d, 3H, *J* = 7.1 Hz, 9-Me), 1.48 (s, 3H, 2-Me), 1.54 (s, 3H, 2-Me), 2.21 (t, 1H, *J* = 5 Hz, OH), 2.76(d, 1H, *J* = 17 Hz, 3-H), 2.78(d, 1H, *J* = 17 Hz, 3-H), 3.69 (m, 2H, 6-CHHOH and 9-H), 3.87 (m, 2H, 6-CHHOH and 6-H), 5.05 (s, 1H, 10-OH), 5.91 (dd, 1H, *J*_1_ = 5 Hz, *J*_2_ = 10 Hz, 7-H), 6.05 (dd, 1H, *J*_1_ = 5 Hz, *J*_2_ = 10 Hz, 8-H), 11.34 (s, 1H, 5-OH), ^13^C-NMR δ (CDCl_3_): 22.81 (6-Me), 26.53 (2-Me), 27.50 (2-Me), 29.26 (C6), 39.12 (C9), 48.83 (C3), 67.79 (9-CH_2_OH), 80.42 (C2), 106.32 (C4a), 121.30 (C5a), 124.06 (C8), 135.01 (C7), 135.28 (C9a), 143.95 (C10), 152.48 (C5), 174.82 (C10a), 198.84 (C4). IR (KBr) 3250, 1641.1 and 1438.8 cm^−1^. M.p. 207–210 °C. Anal. Found: C, 67.20; H, 6.83; Calc. for C_17_H_20_O_5_: C, 67.09; H, 6.62. 5,10-dihydroxy-9-(hydroxymethyl)-2,2,6-trimethyl-2,3,6,9-tetrahydro-4*H*-benzo[*g*]chromen-4-one (**6**), 25.3 mg (0.08 mmol, 20.5% yield): ^1^H-NMR δ (CDCl_3_): 1.31 (d, 3H, *J* = 7 Hz, 6-Me), 1.48 (s, 3H, 2-Me), 1.54 (s, 3H, 2-Me), 2.17 (s, 1H, OH), 2.75(d, 1H, *J* = 17 Hz, 3-H), 2.77(d, 1H, *J* = 17 Hz, 3-H), 3.62(m, 1H, 6-H), 3.72 (m, 1H, 9-H), 3.91 (m, 2H, 9-CH_2_OH), 5.70 (s, 1H, 5-OH), 5.82 (dd, 1H, *J*_1_ = 5 Hz, *J*_2_ = 10 Hz, 7-H), 6.12 (dd, 1H, *J*_1_ = 5 Hz, *J*_2_ = 10 Hz, 8-H), 11.48 (s, 1H, 10-OH), ^13^C-NMR δ (CDCl_3_): 22.86 (9-Me), 26.83 (2-Me), 27.62 (2-Me), 31.12 (C9), 37.53 (C6), 48.78 (C3), 67.57 (5-CH_2_OH), 80.58 (C2), 105.43 (C4a), 115.92 (C5a), 125.39 (C7), 132.39 (C8), 134.69 (C10a), 139.45 (C9a), 143.17 (C10), 151.45 (C5), 197.85 (C4). IR (KBr) 3223.5, 1632.7 and 1349.7 cm^−1^. M.p. 205–207 °C. Anal. Found: C, 67.35; H, 6.83; Calc. for C_17_H_20_O_5_: C, 67.09; H, 6.62. IR (cm^−1^) 3291.5, 2956.5, 1619.8, 1471.1. M.p. 212–213 °C. Anal. Found: C, 72.53; H, 7.19. Calc. for C_19_H_22_O_4_: C, 67.09; H, 6.62.

### 3.2. Computational Calculation

The calculations were carried out using the Gaussian 09 [42] program package. No symmetry constraints were imposed on the optimizations, which were performed at the DFT B3LYP/6-311 ++G (d,p) level. No imaginary vibrational frequencies were found at the optimized geometries, indicating that they are true minima of the potential energy surface. NBO analysis was performed using the NBOPro 6.0 [43] program package. AIM analysis were performed using the AIM2000 [44] program package.

## 4. Conclusions 

A series of synthetic regioisomeric antitumor tricyclic hydroquinones were obtained, structurally characterized, and the multiple IHBs present theoretically studied. The synthesis of compounds **3**, **4**, **5**, and **6** were carried out in a similar way than previously-reported compounds **1** and **2**. Both yields and regioisomeric ratios for **4**–**5** and **5**–**6** were similar to those found in **1**–**2**.

A ranking of the energy involved in the IHB formation between the proton of a phenolic hydroxyl group with an *ortho*-carbonyl group, forming a six-membered ring, in the IHB formation between the oxygen atom of a phenolic hydroxyl group and the proton of an hydroxyalkyl group, forming a seven-membered ring, in the IHB formation between the proton of a phenolic hydroxyl group with the oxygen atom of the hydroxyl group of a hydroxyalkyl group, forming a seven-membered ring and in the IHB between a proton of a phenolic hydroxyl group and an oxygen atom directly bonded to aromatic ring in *ortho* position, forming a five-membered ring, was constructed.

The strongest intramolecular hydrogen bonds are those involving a phenolic proton and carbonyl oxygen atom forming a six-membered ring, and the weakest are those involving a phenolic proton with the oxygen atom of the chromenone ring, which forms five-membered rings.

NBO and AIM analysis allowed us to rationalize, in most cases, the conformational preferences of these molecules in terms of the hyperconjugative donor-acceptor stabilization energies involved in the multiple IHBs present in each molecule. An exception is represented by **3*R***, where steric repulsions seem to predominate on the main rotamer, instead of IHB stabilization energies.

These results can be useful in the design of biologically-active molecules. Additionally, the two pairs of synthesized regioisomeric analogues of biologically-active hydroquinones **1** and **2**, allowed the assessment of the possibility to experimentally access them.

## Figures and Tables

**Table 1:** Boltzmann population for rotamers of **1**.

Compound	E_rel_ (Kcal/mol)	%
**1a**	0.0	59.41
**1b**	0.70	18.12
**1c**	0.57	22.48

**Table 2:** Boltzmann population for rotamers of **2**.

Compound	E_rel_ (Kcal/mol)	%
**2a**	0	99.99
**2b**	7.46	0.00
**2c**	5.31	0.01

**Table 3:** Boltzmann population for rotamers of **3*S***.

Compound	E_rel_ (Kcal/mol)	%
**3*S*a**	0.0	95.33
**3*S*b**	1.88	4.00
**3*S*c**	2.88	0.67

**Table 4:** Boltzmann population for rotamers of **3*R***.

Compound	E_rel_ (Kcal/mol)	%
**3*R*a**	0.87	17.85
**3*R*b**	1.64	4.87
**3*R*c**	0	77.28

**Table 5:** Boltzmann population for rotamers of **4*S***.

Compound	E_rel_ (Kcal/mol)	%
**4*S*a**	0	100.00
**4*S*b**	6.65	0.00
**4*S*c**	6.97	0.00

**Table 6:** Boltzmann population for rotamers of **4*R***.

Compound	E_rel_ (Kcal/mol)	%
**4*R*a**	0	99.99
**4*R*b**	5.42	0.01
**4*R*c**	9.19	0.00

**Table 7:** Boltzmann population for rotamers of **5**.

Compound	E_rel_ (Kcal/mol)	%
**5a**	0.0	48.44
**5b**	0.44	23.30
**5c**	0.32	27.88

**Table 8:** Boltzmann population for rotamers of **6**.

Compound	E_rel_ (Kcal/mol)	%
**6a**	0.0	57.18
**6b**	0.24	38.14
**6c**	1.48	4.69

**Table 9:** Stabilization energies (kcal/mol) for selected NBO pairs (donor-acceptor) given by second-order perturbation energies for **1** and **2**.

Molecule	LP O → σ* H-O		
	O9^…^H-O1′	O1′^…^H-O10	O1^…^H-O9
**1a**	5.38		33.08
**1b**			29.64
**1c**			30.69
**2a**		16.26	30.59

**Table 10:** Atoms-in-molecules parameters for **1** and **2**. Electron density at the critical point ρ^BCP^ (a.u), its Laplacian ∇^2^ρ (a.u.), electron kinetic energy density G (a.u.), ptential energy density V (a.u.), total electron energy density H (a.u.), and hydrogen bond energy E_HB_ (kcal/mol).

Molecule	Bond	ρ^BCP^	∇^2^ρ	G	V	H	E_HB_
**1a**	O9^…^H-O1′	0.0232	−0.0217	0.0198	−0.0450	−0.0252	5.62
	O1^…^H-O9	0.0600	−0.0392	0.0510	−0.1118	−0.0608	19.67
**1b**	O1^…^H-O9	0.0564	−0.0386	0.0483	−0.1063	−0.0580	18.17
**1c**	O1^…^H-O9	0.0576	−0.0389	0.0492	−0.1081	−0.0589	18.67
**2a**	O1′^…^H-O10	0.0217	−0.0211	0.0190	−0.0433	−0.0243	5.27
	O1^…^H-O9	0.0568	−0.0387	0.0486	−0.1069	−0.0583	18.32

**Table 11:** Stabilization energies (kcal/mol) for selected NBO pairs (donor-acceptor) given by second-order perturbation energies for **3** and **4**.

Molecule	LP O → σ* H-O		
	O9^…^H-O1′	O1′^…^H-O10	O1^…^H-O9
**3*S*a**	4.61		33.24
**3*R*a**	8.50		34.92
**3*R*c**			30.73
**4Sa**		20.61	31.04
**4*R*a**		15.67	30.45

**Table 12:** Atoms-in-molecules parameters for **3** and **4**. Electron density at the critical point ρ^BCP^ (a.u), its Laplacian ∇^2^ρ (a.u.), electron kinetic energy density G (a.u.), potential energy density V (a.u.), total electron energy density H (a.u.), and hydrogen bond energy EHB (kcal/mol).

Molecule	Bond	ρ^BCP^	∇^2^ρ	G	V	H	E
**3*S*a**	O9^…^H-O1′	0.0221	−0.0205	0.0187	−0.0425	−0.0238	5.30
	O1^…^H-O9	0.0602	−0.0393	0.0512	−0.1122	−0.0610	19.80
**3*R*a**	O9^…^H-O1′	0.0292	−0.0274	0.0259	−0.0587	−0.0328	7.69
	O1^…^H-O9	0.0620	−0.0396	0.0526	−0.1151	−0.0625	20.55
**3*R*c**	O1^…^H-O9	0.0576	−0.0389	0.0492	−0.1081	−0.0589	18.67
**4*S*a**	O1′^…^H-O10	0.0440	−0.0368	0.0398	−0.0888	−0.0490	13.43
	O1^…^H-O9	0.0578	−0.0389	0.0494	−0.1085	−0.0591	18.76
**4*R*a**	O1′^…^H-O10	0.0385	−0.0344	0.0352	−0.0790	−0.0438	11.33
	O1^…^H-O9	0.0571	−0.0386	0.0487	−0.1071	−0.0584	18.45

**Table 13:** Stabilization energies (kcal/mol) for selected NBO pairs (donor-acceptor) given by second-order perturbation energies for **5** and **6**.

Molecule	LP O → σ* H-O			
	O5^…^H-O1′	O4^…^H-O5	O1^…^H-O10	O10^…^H-O1′
**5a**	4.41	21.57	1.07	
**5b**		19.53	1.09	
**5c**		19.93	1.10	
**6a**		19.86	1.16	4.40
**6b**		19.88	1.06	

**Table 14:** Atoms-in-molecules parameters for **5** and **6**. Electron density at the critical point ρ^BCP^ (a.u), its Laplacian ∇^2^ρ(a.u.), electron kinetic energy density G (a.u.), potential energy density V (a.u.), total electron energy density H (a.u.), and hydrogen bond energy EHB (kcal/mol).

Molecule	Bond	ρ^BCP^	∇^2^ρ	G	V	H	E
**5a**	O5^…^H-O1′	0.0216	−0.0202	0.0183	−0.0417	−0.0234	5.16
	O4^…^H-O5	0.0460	−0.0338	0.0385	−0.0855	−0.0470	13.56
	O1^…^H-O10	-	-	-	-	-	-
**5b**	O4^…^H-O5	0.0436	−0.0331	0.0367	−0.0817	−0.0450	12.65
	O1^…^H-O10	-	-	-	-	-	-
**5c**	O4^…^H-O5	0.0441	−0.0332	0.0371	−0.0825	−0.0454	12.83
	O1^…^H-O10	-	-	-	-	-	-
**6a**	O4^…^H-O5	0.0470	−0.0346	0.0397	−0.0881	−0.0484	14.03
	O1^…^H-O10	-	-	-	-	-	-
	O10^…^H-O1′	0.0350	−0.0316	0.0314	−0.0707	−0.0393	9.82
**6b**	O4^…^H-O5	0.0441	−0.0333	0.0371	−0.0825	−0.0454	12.83
	O1^…^H-O10	-	-	-	-	-	-

## References

[1] Grabowski S.J. (2011). What is the covalency of hydrogen bonding?. Chem. Rev..

[2] Kojic-Prodic B., Molcanov K. (2008). The nature of hydrogen bond: New iinsights into old theories. Acta Chim. Slov..

[3] Kuhn B., Mohr P., Stahl M. (2010). Intramolecular hydrogen bonding in medicinal chemistry. J. Med. Chem..

[4] Castellano R.K. (2014). Special issue: Intramolecular hydrogen bonding. Molecules.

[5] Scheiner S. (2016). Assessment of the presence and strength of H-bonds by means of corrected NMR. Molecules.

[6] Bilonda M.K., Mammino L. (2017). Intramolecular hydrogen bonds in conformers of quinine and quinidine: An HF, MP2 and DFT study. Molecules.

[7] Nagy P.I. (2014). Competing intramolecular vs. Intermolecular hydrogen bonds in solution. Int. J. Mol. Sci..

[8] Sobczyk L., Chudoba D., Tolstoy P.M., Filarowski A. (2016). Some brief notes on theoretical and experimental investigations of intramolecular hydrogen bonding. Molecules.

[9] Tessensohn M.E., Webster R.D. (2016). Using voltammetry to measure hydrogen-bonding interactions in non-aqueous solvents: A mini-review. Electrochem. Commun..

[10] Abdel-Lateff A., Konig G.M., Fisch K.M., Holler U., Jones P.G., Wright A.D. (2002). New antioxidant hydroquinone derivatives from the algicolous marine fungus *Acremonium* sp.. J. Nat. Prod..

[11] Aknin M., Dayan T.L.A., Rudi A., Kashman Y., Gaydou E.M. (1999). Hydroquinone antioxidants from the indian ocean tunicate aplidium savignyi. J. Agric. Food Chem..

[12] Shen Y.C., Chen C.Y., Kuo Y.H. (2001). New sesquiterpene hydroquinones from a taiwanese marine sponge, hippospongia metachromia. J. Nat. Prod..

[13] Marais J.P.J., Mueller-Harvey I., Brandt E.V., Ferreira D. (2000). Polyphenols, condensed tannins, and other natural products in onobrychis viciifolia (sainfoin). J. Agric. Food Chem..

[14] Schieber A., Keller P., Carle R. (2001). Determination of phenolic acids and flavonoids of apple and pear by high-performance liquid chromatography. J. Chromatogr. A.

[15] Parejo I., Viladomat F., Bastida J., Codina C. (2001). A single extraction step in the quantitative analysis of arbutin in bearberry (arctostaphylos uva-ursi) leaves by high-performance liquid chromatography. Phytochem. Anal..

[16] Monks T.J., Hanzlik R.P., Cohen G.M., Ross D., Graham D.G. (1992). Quinone chemistry and toxicity. Toxicol. Appl. Pharm..

[17] Valgimigli L., Amorati R., Fumo M.G., DiLabio G.A., Pedulli G.F., Ingold K.U., Pratt D.A. (2008). The unusual reaction of semiquinone radicals with molecular oxygen. J. Org. Chem..

[18] Song Y., Buettner G.R. (2010). Thermodynamic and kinetic considerations for the reaction of semiquinone radicals to form superoxide and hydrogen peroxide. Free Radic. Biol. Med..

[19] Okun J.G., Lummen P., Brandt U. (1999). Three classes of inhibitors share a common binding domain in mitochondrial complex i (nadh : Ubiquinone oxidoreductase). J. Biol. Chem..

[20] Urra F.A., Weiss-Lopez B., Araya-Maturana R. (2016). Determinants of anti-cancer effect of mitochondrial electron transport chain inhibitors: Bioenergetic profile and metabolic flexibility of cancer cells. Curr. Pharm. Des..

[21] Urra F.A., Córdova-Delgado M., Lapier M., Orellana-Manzano A., Acevedo-Arévalo L., Pessoa-Mahana H., González-Vivanco J.M., Martínez-Cifuentes M., Ramírez-Rodróguez O., Millas-Vargas J.P. (2016). Small structural changes on a hydroquinone scaffold determine the complex i inhibition or uncoupling of tumoral oxidative phosphorylation. Toxicol. Appl. Pharmacol..

[22] Inbaraj J.J., Chignell C.F. (2004). Cytotoxic action of juglone and plumbagin: A mechanistic study using hacat keratinocytes. Chem. Res. Toxicol..

[23] Schreiber J., Mottley C., Sinha B.K., Kalyanaraman B., Mason R.P. (1987). One-electron reduction of daunomycin, daunomycinone, and 7-deoxydaunomycinone by the xanthine/xanthine oxidase system: Detection of semiquinone free radicals by electron spin resonance. J. Am. Chem. Soc..

[24] Armendariz-Vidales G., Martinez-Gonzalez E., Cuevas-Fernandez H.J., Fernandez-Campos D.O., Burgos-Castillo R.C., Frontana C. (2013). The stabilizing role of intramolecular hydrogen bonding in disubstituted hydroxy-quinones. Electrochim. Acta.

[25] Salazar R., Vidal J., Martínez-Cifuentes M., Araya-Maturana R., Ramírez-Rodríguez O. (2015). Electrochemical characterization of hydroquinone derivatives with different substituents in acetonitrile. New J. Chem..

[26] Foti M.C., Johnson E.R., Vinqvist M.R., Wright J.S., Barclay L.R.C., Ingold K.U. (2002). Naphthalene diols: A new class of antioxidants intramolecular hydrogen bonding in catechols, naphthalene diols, and their aryloxyl radicals. J. Org. Chem..

[27] Rodriguez J., Olea-Azar C., Cavieres C., Norambuena E., Delgado-Castro T., Soto-Delgado J., Araya-Maturana R. (2007). Antioxidant properties and free radical-scavenging reactivity of a family of hydroxynaphthalenones and dihydroxyanthracenones. Bioorg. Med. Chem..

[28] Lown J.W. (1985). Molecular mechanisms of action of anticancer agents involving free radical intermediates. Adv. Free Radic. Biol. Med..

[29] Pedroza D.A., De Leon F., Varela-Ramirez A., Lema C., Aguilera R.J., Mito S. (2014). The cytotoxic effect of 2-acylated-1,4-naphthohydroquinones on leukemia/lymphoma cells. Bioorg. Med. Chem..

[30] Yang J.T., Li Z.L., Wu J.Y., Lu F.J., Chen C.H. (2014). An oxidative stress mechanism of shikonin in human glioma cells. PLoS ONE.

[31] Loya S., Bakhanashvili M., Kashman Y., Hizi A. (1995). Peyssonol-a and peyssonal-b, 2 novel inhibitors of the reverse transcriptases of human-immunodeficiency-virus type-1 and type-2. Arch. Biochem. Biophys..

[32] Araya-Maturana R., Cardona W., Cassels B.K., Delgado-Castro T., Ferreira J., Miranda D., Pavani M., Pessoa-Mahana H., Soto-Delgado J., Weiss-Lopez B. (2006). Effects of 9,10-dihydroxy-4,4-dimethyl-5,8-dihydro-1(4*H*)-anthracenone derivatives on tumor cell respiration. Bioorg. Med. Chem..

[33] Araya-Maturana R., Delgado-Castro T., Garate M., Ferreira J., Pavani M., Pessoa-Mahana H., Cassels B.K. (2002). Effects of 4,4-dimethyl-5,8-dihydroxynaphtalene-1-one and 4,4-dimethyl-5,8-dihydroxytetralone derivatives on tumor cell respiration. Bioorg. Med. Chem..

[34] Urra F.A., Martínez-Cifuentes M., Pavani M., Lapier M., Jaña-Prado F., Parra E., Maya J.D., Pessoa-Mahana H., Ferreira J., Araya-Maturana R. (2013). An *ortho*-carbonyl substituted hydroquinone derivative is an anticancer agent that acts by inhibiting mitochondrial bioenergetics and by inducing g2/m-phase arrest in mammary adenocarcinoma ta3. Toxicol. Appl. Pharmacol..

[35] Soto-Delgado J., Bahamonde-Padilla V., Araya-Maturana R., Weiss-Lopez B.E. (2013). On the mechanism of biological activity of hydroquinone derivatives that inhibit tumor cell respiration. A theoretical study. Comp. Theor. Chem..

[36] Dobado J.A., Gomez-Tamayo J.C., Calvo-Flores F.G., Martinez-Garcia H., Cardona W., Weiss-Lopez B., Ramirez-Rodriguez O., Pessoa-Mahana H., Araya-Maturana R. (2011). Nmr assignment in regioisomeric hydroquinones. Magn. Reson. Chem..

[37] Araya-Maturana R., Cassels B.K., Delgado-Castro T., Valderrama J.A., Weiss-Lopez B.E. (1999). Regioselectivity in the diels-alder reaction of 8,8-dimethylnaphthalene-1,4,5(8*H*)-trione with 2,4-hexadien-1-ol. Tetrahedron.

[38] Sethna S.M. (1951). The elbs persulfate oxidation. Chem. Rev..

[39] Araya-Maturana R., Cassels B.K., Delgado-Castro T., Hurtado-Guzmán C., Jullian C. (1999). Complete assignment of the 13c nmr spectra of a series of 5,8-disubstituted 4,4-dimethylanthracene-1,9,10(4*H*)-triones. Magn. Reson. Chem..

[40] Martínez-Cifuentes M., Weiss-López B.E., Santos L.S., Araya-Maturana R. (2014). Intramolecular hydrogen bond in biologically active o-carbonyl hydroquinones. Molecules.

[41] Espinosa E., Molins E., Lecomte C. (1998). Hydrogen bond strengths revealed by topological analyses of experimentally observed electron densities. Chem. Phys. Lett..

[42] Frisch M.J., Trucks G.W., Schlegel H.B., Scuseria G.E., Robb M.A., Cheeseman J.R., Scalmani G., Barone V., Mennucci B., Petersson G.A. (2009). Gaussian 09.

[43] Glendening E.D., Badenhoop J.K., Reed A.E., Carpenter J.E., Bohmann J.A., Morales C.M., Landis C.R., Weinhold F. (2013). Nbo 6.0.

[44] Biegler-Konig F., Schonbohm J. (2002). Update of the aim 2000-program for atoms in molecules. J. Comp. Chem..

